# Novel endoscopic approach for duodenal neuroendocrine tumors: partial-closure-assisted endoscopic submucosal resection with a ligation device

**DOI:** 10.1055/a-2781-6157

**Published:** 2026-02-13

**Authors:** Yuichi Hirano, Shunsuke Yoshii, Tomoki Michida, Ryu Ishihara

**Affiliations:** 1 53312 Department of Gastrointestinal Oncology, Osaka International Cancer Institute, Osaka, Japan

Endoscopic resection is recommended for small non-functional duodenal neuroendocrine tumors (NETs 1 ). Endoscopic submucosal resection with a ligation device (ESMR-L) is one of the commonly used techniques; however, in the thin-walled duodenum, unintended full-thickness resection may occur 2 , and the resulting perforation can be severe. Herein, we present two cases of duodenal NETs treated with a novel strategy combining ESMR-L with pre-emptive partial closure (partial-closure-assisted ESMR-L [PC-ESMR-L]; Video 1 ).

A novel endoscopic approach for duodenal NETs: partial-closure-assisted ESMR-L (PC-ESMR-L). ESMR-L, endoscopic submucosal resection with a ligation device; NET, neuroendocrine tumor.Video 1

**Fig. 1:**
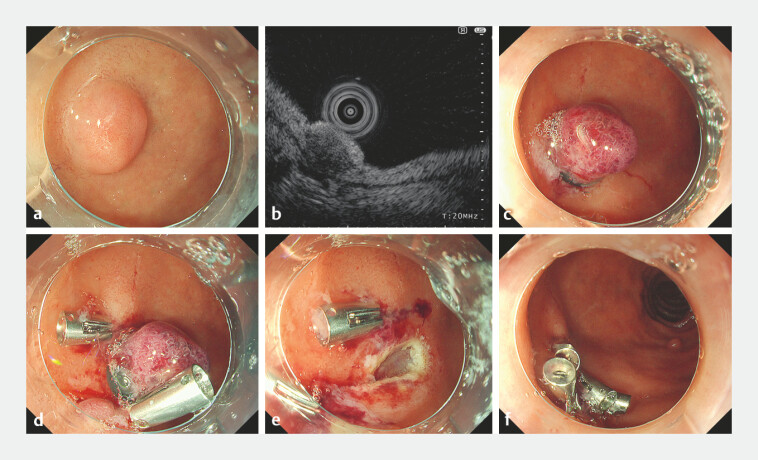
Endoscopic images of case 1. **a** A dome-shaped, 7-mm elevated lesion on the anterior duodenal bulb. **b** An endoscopic ultrasound image using a 20-MHz miniature probe. **c** A band ligation of the tumor. **d** Clips on both sides of the lesion prior to resection. **e** The post-resection defect. **f** Complete closure using additional clips.

**Fig. 2:**
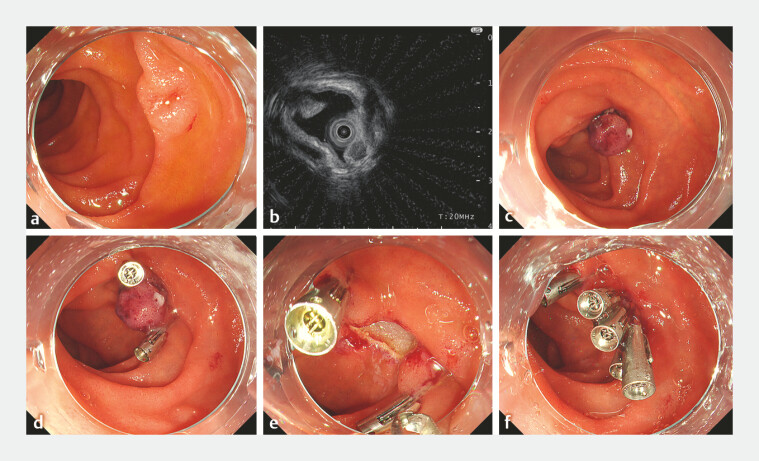
Endoscopic images of case 2. **a** A 6-mm elevated lesion with central depression on the anterior wall of the descending duodenum. **b** An endoscopic ultrasound image using a 20-MHz miniature probe. **c** A band ligation of the tumor. **d** Clips on both sides of the lesion prior to resection. **e** The post-resection defect. **f** Complete closure using additional clips.

In the first case, a 78-year-old man had a 7-mm elevated lesion on the anterior duodenal bulb, and endoscopic ultrasonography showed a hypoechoic lesion extending into the deep submucosa accompanied by thinning of the submucosal layer ( Fig. 1 **a, b** ). In ESMR-L, to anticipate difficulty in closing an unexpected full-thickness defect, two clips were placed on both sides of each lesion immediately after band ligation ( Fig. 1 **c, d** ). By firmly suctioning the duodenal wall into the clip jaws, the wall was folded in a mountain-like configuration. This maneuver converted the expected circular post-resection defect into an elongated rugby-ball- or slit-like shape, allowing easy and complete closure ( Fig. 1 **e, f** ). The second case, a 59-year-old man, had a 6-mm lesion on the anterior wall of the second portion and he underwent resection using the same technique as in the first case ( Fig. 2 ). Both patients recovered uneventfully, and pathology showed NET G1 with negative resection margins.

In PC-ESMR-L, partial closure of the lesion margins with clips before resection may increase the thickness of the submucosal layer by plication of the duodenal wall, which could potentially reduce the risk of perforation during resection. When resection results in a full-thickness defect, loss of muscular and serosal support may allow the defect to widen under wall tension and intraluminal pressure. PC-ESMR-L helps maintain a narrow defect and facilitates secure closure without specialized devices or additional cost, potentially enabling safer, margin-secure resection.

Endoscopy_UCTN_Code_CCL_1AB_2AZ_3AB Endoscopy_UCTN_Code_TTT_1AO_2AG_3B
